# Identification of Antifungal Targets Based on Computer Modeling

**DOI:** 10.3390/jof4030081

**Published:** 2018-07-04

**Authors:** Elena Bencurova, Shishir K. Gupta, Edita Sarukhanyan, Thomas Dandekar

**Affiliations:** 1 Department of Bioinformatics, Am Hubland, Biozentrum, University of Würzburg, 97074 Würzburg, Germany; elena.bencurova@uni-wuerzburg.de (E.B.); shishir.gupta@uni-wuerzburg.d (S.K.G.); edita.sarukhanyan@uni-wuerzburg.de (E.S.); 2 BioComputing Unit, EMBL Heidelberg, 69117 Heidelberg, Germany

**Keywords:** *Aspergillus*, metabolic pathways, computational modelling, drug design

## Abstract

*Aspergillus fumigatus* is a saprophytic, cosmopolitan fungus that attacks patients with a weak immune system. A rational solution against fungal infection aims to manipulate fungal metabolism or to block enzymes essential for *Aspergillus* survival. Here we discuss and compare different bioinformatics approaches to analyze possible targeting strategies on fungal-unique pathways. For instance, phylogenetic analysis reveals fungal targets, while domain analysis allows us to spot minor differences in protein composition between the host and fungi. Moreover, protein networks between host and fungi can be systematically compared by looking at orthologs and exploiting information from host–pathogen interaction databases. Further data—such as knowledge of a three-dimensional structure, gene expression data, or information from calculated metabolic fluxes—refine the search and rapidly put a focus on the best targets for antimycotics. We analyzed several of the best targets for application to structure-based drug design. Finally, we discuss general advantages and limitations in identification of unique fungal pathways and protein targets when applying bioinformatics tools.

## 1. Introduction

In the last 40 years, fungal infections have been considered to be of the most common human diseases [1]. Incidence of invasive fungal diseases is rising with higher numbers of organ transplantations, medical interventions, chronic granulomatous disease, acute leukemia, and AIDS [2,3]. Fungal spores are everywhere: Fungi are a natural part of the environment that occupy every possible niche including soil, air, water, and even hospitals. Moreover, fungi are emerging pathogens [4]. For instance, *Candida auris,* first described in Japan in 2009 is currently spread in 17 countries on 5 continents, is drug resistant, and is a potential threat to the public health [5]. In this review, we focus on *Aspergillus fumigatus*—a major fungal pathogen and other invasive fungal species, such as *Candida albicans*. *A. fumigatus* is an excellent model organism to study severe fungal infections. The infection first begins at respiratory pathways and then spreads by sepsis. Prone to an antibiotic attack include the metabolism, and the cell wall. In the following chapters, we summarize and exemplify how bioinformatics methods can identify potential drug targets, rank them, and help to target the best as specific as possible by drugs.

## 2. Fungal Diseases and Domain Analysis

Fungal infections represent a serious health problem worldwide. While superficial infection, caused by dermatophytes, occurs in approximately 25% of the general population [6], invasive fungal infections are less common. These, however, are often associated with a high mortality rate. The majority of fungal-related deaths are caused by the four genera: *Cryptococcus*, *Candida*, *Pneumocystis*, and *Aspergillus*. *Aspergillus* causes several types of infection: (i) chronic pulmonary aspergillosis, (ii) an invasive aspergillosis, and (iii) it is a strong aeroallergen, causing allergies and asthma (reviewed in [7]). Among the several hundred *Aspergillus* species, only a few of them are able to infect man, including *A. fumigatus* and *A. flavus* (reviewed in [8]). The most effective medications against human aspergillosis are polyenes, azoles, and echinocandins, however, their toxicity, interactions with other drugs, and increased drug resistance of several *Aspergillus* strains are the main reasons to persistently seek novel treatments/therapies/antidotes.

The fight against eukaryotic pathogens is especially challenging since all Eukaryotes have similar types of cells with a nucleus and big ribosomes, and the same metabolic and regulatory pathways as their mammalian host. Not only the treatment of fungal diseases challenging, but also other infections caused by Eukaryotes, e.g., Apicomplexa such as *Plasmodium*, *Myriospora*, and *Toxoplasma*, where the treatment with standard antibiotics does not work. One strategy is to target proteins present in both the eukaryotic pathogen and its host, but aiming at differences in domain structure so that the drug affects mainly the protein in the pathogen. Zirkel et al. combined in silico modelling and PCR data to demonstrate that methylene blue selectively inhibits glutathione reductase of *Plasmodium falciparum* without affecting the human glutathione reductase much [9]. Pharmacologically interesting CA/C1 peptidases have also shown several functional overlaps in *P. falciparum*, *P. berghei*, and their hosts—*Mus musculus* and *Homo sapiens*. Domain and regulation differences allow us to specifically target cathepsins in plasmodia in critical stages of their life cycle [10].

Fungal parasites target various animal species (Table 1). Mycoses in veterinary research are rarely reported, as diagnosis is difficult. However, they can induce a serious multiple organ failure. Due to changes in climate that promote the global spread and proliferation/expansion of microorganisms, pathogenic fungi pose a significant threat as an emerging health hazard [4]. On the other hand, several animal models have been established for investigation of fungal pathogenesis, host–fungi interaction, and development of antifungal drugs. For example, mice, rabbits, guinea pigs, chickens, and ducks represent such models for the study of aspergillosis. Despite plenty of benefits, their size, phenotypic deviation, and genetic homogeneity of inbred strains are limiting factors when (trying to mirror) mimicking the clinical situation in humans [11].

**Figure 1:**
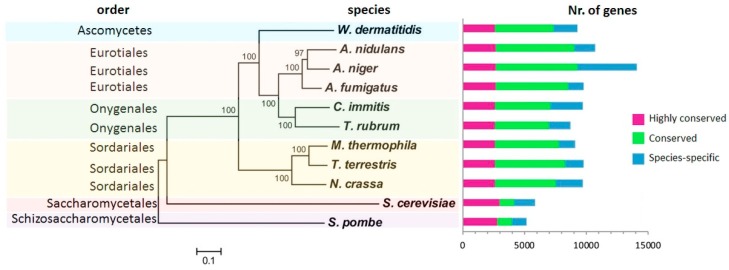
Comparison of the gene conservation and their phylogenetic proximity among selected fungal species. The phylogenetic tree was reconstructed using the MUSCLE alignment based on more than 2000 single copy genes. The number of highly conserved genes found in each species is depicted in magenta, the genes conserved in at least two species are marked in green and the unique genes are colored in sky blue (modified from [13]).

*Aspergillus fumigatus* can be used as model organism for study of ascomycetous pathogens. The tendencies in current research are aimed to develop an antimycotic drug that will treat the immuno-compromised state caused by the infection with *A. fumigatus*, *C. albicans*, *Cryptococcus*, and emerging molds of the order Mucorales [12]. The genomes of important fungal pathogens are already known, including *A. fumigatus*, *C. neoformans*, *C. albicans*, *Parasitella parasitica*, *Histoplasma capsulatum*, and *Pneumocystis jirovecii.* Despite the significant diversity in fungal genomes, several studies/publications revealed stress specific gene conservation among fungal species. Figure 1 exemplifies conserved genes (in magenta) in several fungi and important pathogens. Highly conserved genes were found mostly among transmembrane proteins, e.g., diverse transporter families, acyltransferase proteins, and phosphotransferases (tyrosine; serine/threonine and aminoglycoside). Proteins such as chitin synthase of *W. dermatitidis* (highlighted in sky blue in Figure 1), were found only in one type of the species and can serve as candidates for the species-specific treatment [13]. Liu and colleagues identified 240 unique conserved genes among the 10 fungal species via the comparison of the genome [14]. They found that only 43 of them shared less than 40% identity with human genes. Analogous to this work, Coronado et al. [15] revealed 187 cell wall-related proteins in Saccharomyces cerevisiae which were compared with the 17 other fungal genomes, including *A. fumigatus*, *C. neoformans*, and *C. albicans*. While the group of adhesins and invasins were conserved only in the species closely related to the species Saccharomyces, glycosylases, aspartyl proteases, and proteins involved in plant pathogenesis showed conserved patterns among all the tested species [15]. The extensive study of Fedorova et al. focused on the genome comparison of *A. fumigatus*, *N. fischeri*, and *A. clavatus* [16]. It showed the presence of several large species-specific and isolate-specific chromosomal islands that probably contribute to rapid adaptation from saprophytic to parasitic lifestyle of *Aspergillus.*

**Figure 2:**
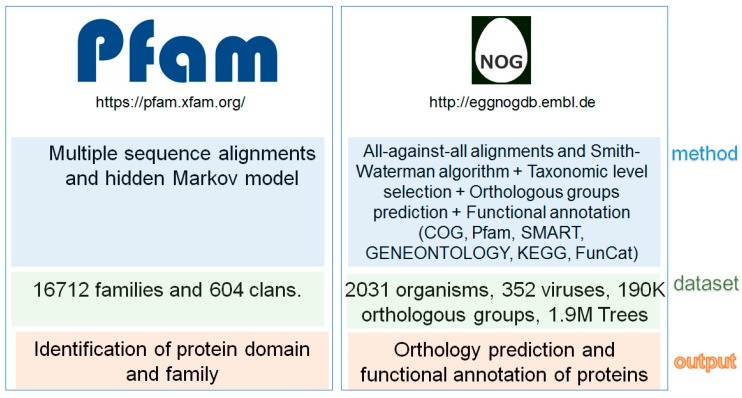
Comparison between Pfam and EggNog databases.

Genomic and proteomic analysis is crucial for understanding fungal pathogenesis. Data obtained from the omics analysis are essential for the annotation processes that lead to the recognition of the functional domains and motifs of proteins. Several databases were established to study the protein domains. The most popular is Pfam (Figure 2), listing more than 16,000 protein families [17]. The concept of the Pfam is simple: each entry is described by multiple alignments, and the suitable alignment is retrieved by a sequence or key word query: The seed alignment contains a narrow set of representative members of a family. Subsequently, the hidden Markov model builds the full alignment, which includes all protein sequences of the protein family [18]. Pfam contains two divisions: (i) Pfam-A is the manually curated part covering 16,712 families (Pfam 31.0) and (ii) Pfam-B, which consists of automatically generated clusters produced by an automatic domain decomposition algorithm (ADDA). A similar approach was implemented for other protein family and domain databases—such as SMART, ProDom, and TIGRFAM—that also use multiple sequence alignment and profile-hidden Markov models. The SMART database is synchronized with UniProt [19], Ensembl [20] and STRING databases [21]. SMART contains more than 1300 manually curated protein domains [22], while the ProDom [23] contains the set of the domain families generated only from UniProt. The information about the microbial proteins is collected in the TIGRFAM (www.jcvi.org/cgi-bin/tigrfams/index.cgi), which contains the protein families hierarchically organized based on their functional role. Compared to the other databases, TIGRFAM should only hit equivalogs (protein families conserved in function since their last common ancestor).

The Clusters of Orthologous Groups (COGs) database (http://www.ncbi.nlm.nih.gov/COG/) is based on the hypothesis that orthologs share the same architecture, the three-dimensional structure of the domains, biochemical and cellular functions, as well as the evolutionary origin, which allows the study of the poorly annotated organism. COGs started in 1997 [24] as a project including full-length proteins from five bacterial genomes, one archaeal and one eukaryotic. The analytic method used for COG is based on the search of triangles of bidirectional best hits to assign the orthologs and paralogs and accordingly to estimate and separate the paralogs. COGs uses family-based approach when the whole family is characterized based on the individual member of the whole family, ergo the whole protein family shares the function(s) described for the best-characterized member of the family. The process of the annotation was manually curated for each COG [25].

Despite the worldwide success of the COG database, due to a number of limitations, the COG is no longer considered to be the leader/first choice/most used in evolution-based protein classification. The main drawback of the COG database is the lack of phylogenetic resolution and continuous update. The second limitation is the number of available genomes, which is relatively small (66 genomes, consisting of 138,458 proteins before the implementation of the EggNOG database [26]. This led to the development of the EggNOG database (evolutionary genealogy of genes: non-supervised orthologous groups, Figure 2), founded by European Molecular Biology Laboratory (EMBL) (http://eggnogdb.embl.de). EggNOG uses an approach for the annotation of the orthologous group identical to COG. In the first phase, the orthologous groups are constructed using the Smith–Waterman alignments. Thereafter, groups are identified based on the reciprocal best matches and triangular linkage clustering. Compared to COG, EggNOG is completely automatic, the number of the genes and genomes is significantly higher, and a hierarchy of orthologous groups is created [27]. Currently, COG is implemented in the EggNOG database.

Correct and precise protein annotation is important together with the charting of the evolutionary origin of the proteins. Both help in the identification of novel drug targets. Popular jump-start approaches using those strategies are network-based approaches. In the last 10 years, the steady flow of articles related to network-based strategies increased as a result of several aspects: easy accessibility of the input data, time and cost effectivity, and use of the multi-omics data to optimize the research process. Those strategies offer complete insight into the pathogenesis and treatment of the disease, such as prediction of novel protein targets and drugs [28], characterization of the disease module [29] and identification of the disease pathway for maximizing the effectivity of the pharmaceutical component [30,31].

Three major network-based strategies speed up drug discovery: analysis of regulatory networks, metabolic networks, and protein–protein interaction networks. Regulatory networks are reconstructed from the transcriptome data and help to analyze the regulatory interaction of the genes involved in the pathogenesis. Several computational methods were established to model the regulatory mechanism under physiological and pathological conditions, such as a Bayesian network [32] and a Boolean network [33]. In contrast to regulatory networks, metabolic networks can be reconstructed by several methods that analyze the steady-state flux distributions among the genes associated with the network. The elementary flux modes, convex basis-based pathway concepts, flux balance analysis and choke point analyses are required/crucial in order to reconstruct the metabolic network related to the disease. The most interactive metabolic proteins found in such a network usually correlate with a high number of functional associations and have a significant role in pathogenesis [34,35]. The protein–protein interaction analysis is focused on the interactome data. The reconstruction of the PPI network can be performed with software tools such as R/Bioconductor (http://www.bioconductor.org) and Cytoscape (http://www.cytoscape.org). The analysis of the PPI network consists of protein ranking based on their connectivity and location. Drugs that target central network nodes generally have lethal consequences for a pathogen or targeted cells, while the drugs targeting neighbors of central nodes substantially manipulate the corresponding metabolic pathway [36].

**Figure 3:**
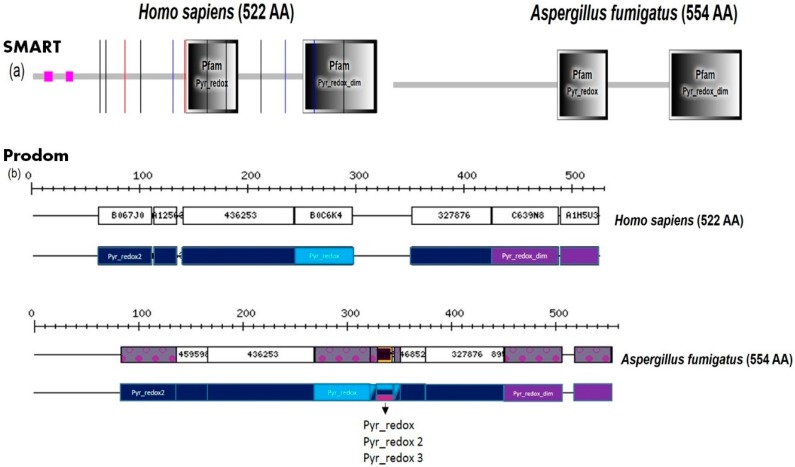
Protein domain analysis of the glutathione reductase showing the similarities between human (UniProt ID: P00390) and *A. fumigatus* protein (UniProt ID: A0A0J5PGY5). Panel (**a**) Both proteins contain a Pyridine nucleotide-disulphide oxidoreductase domain (Pyr_redox; position 233–314 in human, position 261–341 in *Aspergillus*) and the Pyridine nucleotide-disulphide oxidoreductase, dimerization domain (Pyr_redox_dim; position 411–522 in human, position 439–554 in *Aspergillus*). Magenta square: low complexity region, black line: intron (phase 0), blue line: intron (phase 1), red line: intron (phase 2). The analysis was performed by SMART database; Panel (**b**) Domain mapping performed by ProDom database. We enlarged the standard output so that now the specific minor differences in exact domain composition and position of low complexity regions and other features stand out.

The identification and comparison of the ortholog groups in host and pathogen is important to reveal unique differences among the species. Figure 3 illustrates how to scrutinize the protein domain annotation to spot targetable differences. The glutathione reductase from both humans and *A. fumigatus* is analyzed by using two different databases. The domain composition is similar in host and fungus, however, there are several specific differences. There are four domains in both: besides two cofactor-binding domains and the catalytic domain of glutathione reductase, a dimerization domain is present. Main differences between the proteins concern—in particular—the spacing of the domains with respect to each other, localization of low complexity regions, and minor differences in exact domain composition (Figure 3, bottom). These differences could all in principle be targeted and lead to different responses against suitable drugs targeting these. Interestingly, the dimerization domain can be targeted with methylene blue, leading to inactivation of the glutathione reductase. However, the apparent minor differences in domain composition and the resulting overall minor structure differences imply more detrimental effects for the pathogen if methylene blue acts on the protein. The strategy of utilizing a differential effect of methylene blue on the pathogen through and by targeting the same protein in host and pathogen and exploiting the small differences in detailed domain composition has been used previously in malaria as well as *Candida* research [37,38].

Both of the databases provide complementary information, for instance, SMART depicts besides the domain annotation also splicing junctions for human genes (as red, blue, and black thin lines) as well as the low complexity regions. Instead of the annotation function, SMART is linked to other databases such as STRING (prediction of functional interacting partners), KEGG (visualization of a metabolic pathways), PTMcode (posttranslational modification prediction), and eggNOG (database of orthologous genes). In contrast to SMART, Prodom provides more information regarding the exact domain content and classification (graphical representation of the family), allows comparison of several proteins by Blast-P and Blast-X, and is linked with STRAP tools to align and build the phylogenetic tree of family based on their structure.

The following chapters focus on finding host–pathogen differences in a systematic manner, by looking at host–pathogen interaction networks (Chapter 3). We then give bioinformatics details on approaches to evaluate the structures of the identified targets and how to design suitable drugs (Chapters 4 and 5).

## 3. Screens and Counter-Screens for Unique Fungal Protein Targets Using Protein–Protein Interaction Networks and Metabolic Networks

### 3.1. Interactome Based Approaches

Biological networks can help to improve our understanding of biological systems and the communication flow within them. Protein–protein interaction networks (PPIN) are directly applicable for drug discovery. They can be used to identify promising drug targets, taking into account proteins and interactions specific for the pathogen and through which hence the host and the host-specific network is not harmed. Large-scale interspecies host–pathogen protein networks (HP-PPIN) are moreover an important foundation for the understanding of the ways how pathogens interact, invade, and seize control of the human host. Pathogenic proteins tend to interact mainly with hubs and bottlenecks in the PPIN [39,40]. The connectivity in the host–pathogen interactome is directly related to the pathogen fitness during infection [41]. Therefore, high degree proteins of the pathogen in HP-PPIN are promising drug targets. The wiring of PPIN can be determined by a variety of binary and co-complex methods, however, these methods are not suitable for genome-scale prediction of HP-PPIN. Nevertheless, experimental high throughput assays typically include a high number of false positive protein–protein interactions [42] so that there is an enduring need for efficient computational methods to scrutinize and counter-screen existing experimental data. Such methods include the interolog method [43], domain information [44], gene ontology (GO) annotations [45], and information about cellular localization [46,47]. Demonstrating such features in a graphical representation of the interaction network provides an intuitive overview and useful insights to help and analyze complicated relations between host and pathogen [48,49,50]. For instance, the *A. fumigatus*-human and *C. albicans*–human host–pathogen protein–protein interaction network was determined starting from human and yeast intraspecies networks and looking at sequence similar proteins in these fungi with similar interactions with the host [48]. Such a network represents all predicted host–pathogen interactions which were found to be conserved by sequence conservation. To determine the best drug targets in pathogens from such a network, it is necessary to analyze the network further, in particular in terms of topology of the proteins in the network and function of interacting host-proteins.

In this pursuit, the availability of the sequenced genome of *A. fumigatus* [51] has paved the way to discover drug targets through network analysis [28]. Topological analysis of the *A. fumigatus* interactome reveals several hubs and bottlenecks. Because of the higher connectivity compared to other proteins in the network, hubs are often involved in multiple functions, while connecting proteins, so-called bottlenecks, are important for the flow of information among different functional modules. Consequently, deletion of a hub protein or a connecting bottleneck protein can be lethal for the fungus. The top 20% of network proteins based on (hub) degree rank and (bottleneck connectivity rank can be considered to be functionally important, and re-ranking of protein “iP” can be calculated based on HBrank(iP) that gives both ranks equal weight

$$\mathrm{HBrank}\left(\mathrm{iP}\right)={\mathrm{Rank}}_{\mathrm{de}}\left(\mathrm{iP}\right)+{\mathrm{Rank}}_{\mathrm{be}}\left(\mathrm{iP}\right)$$

Here “iP” indicates the ith protein in the reconstructed PPI, Rank_de_ is the ranking of protein “iP” based on degree score and Rank_be_ is the ranking of proteins “iP” based on the “betweenness” score (i.e., the number of interconnected proteins).

The biggest challenge for computational prediction of the interactome is the lack of available verified interactions and the relevant annotation information in most of the pathogens. Therefore, several approaches have been developed to increase the interaction confidence including domain–domain interactions (DDIs) [52], subcellular localization [46], and GO annotation [45] filters. Domain–domain interactions have been used by several authors as an approach to better select relevant PPIs. However, DDIs can also be used to filter interolog interactions [52]. This ensures the most reliable interolog interactions that are supported by at least one experimentally verified PPI. For domain mapping Pfam [53] and InterPro [54] are the best sources, while DOMINE [55] and DIMA 3.0 [56] are good resources to find information of interacting domains according to crystallized structures.

To determine a robust orthology relationship, InParanoid [57,58] or OrthoMCL [59] can be used. Experimentally verified PPIs can be obtained from highly curated databases such as the Database of Interacting Proteins [60]. Several other databases also contain experimental verified PPIs: String [21], BioGrid [61] and common interface resources such as PSICQUIC [62]. The latter can also be used to derive a template for the mapping of putative PPIs.

Similar subcellular localization of two interacting proteins ensures their chances to meet. Moreover, multiple localized proteins increase the possibility of protein interaction with different localized partners. WoLF PSORT [63], KnowPred [64], and YLoc [65] are robust tools to determine the subcellular localization of eukaryotic pathogenic proteins. Likewise, similar GO-annotations of two proteins also indicate their co-involvement in the same biological function of molecular processes. Therefore, to determine this, GO database (The Gene Ontology, 2017), David [66], and a platform such as Blast2GO [67] are a good choice. The network can be constructed with Cytoscape [68] with different plugins (http://apps.cytoscape.org) for further detailed network analysis.

Several topologically important proteins that also participate in *A. fumigatus* metabolism were identified as potential antimycotic targets, such as 1,3-β-glucanosyltransferase (Gel2, AFUA_6G11390) and phosphate transporter (Pho88, AFUA_5G01960) [28].

### 3.2. Metabolic Modeling Approach

Another reliable method to systematically identify drug targets of *A. fumigatus* relies on the fungal metabolism [28]. This includes direct metabolic network modeling using elementary mode analysis (EMA) and flux estimates using gene expression data. One can combine transcriptome data with EMA, looking at high level expression, central position in the metabolism, and the resulting (calculated) strong and central metabolic flux as detailed later in the manuscript. This approach allows us to target metabolic enzymes of the fungus after transcriptome analysis and search for the condition-specific highly expressed enzymes during important phases of the infection.

Metabolic network modeling is used to predict and analyze the flux distribution of different physiological conditions in biological systems [69]. Metabolic processes are a key component of the factors that determine the dynamics and virulence of the pathogens. In the host–pathogen interaction, many interactions rely on metabolic processes. This includes the competition for common resources, exchange of nutrients, and chemical communications. Metabolic networks can be reconstructed for the pathogen on both the small-scale and genome scale. Small-scale reconstruction focuses on a specific metabolic pathway network. For instance, the central carbohydrate metabolism may be easily calculated, pointing out central enzymes involved in many pathways or carrying a lot of flux. Hereby, some previous knowledge or bioinformatics data or biochemical data can help to reveal promising targets in central fungal pathways. In contrast, genome-scale reconstruction requires the complete genome sequence and refined annotation of all the reactions and biomass data [70]. This is very time-consuming and labor-intensive, however, the sum of calculations then directly points out the best targets in pathogens. Thus, Lee and coworkers reconstructed a model of *Staphylococcus aureus* and identified various antimicrobial drug targets [71]. The authors constructed the metabolic network using the genome annotation, functional-pathway analysis, and comparative genomic approaches and finally performed flux balance analysis (FBA)-based in silico single and double gene deletion experiments to identify novel drug targets. Similarly, to analyze fungal drug targets comprehensively, genome-scale metabolic network of *A. fumigatus* can be reconstructed [28]. Reconstruction means starting from biochemical databases to get a full list of all involved enzymes, and critically reexamining this list taking genome annotation, domain, and sequence analysis into account. Otherwise, one cannot be sure on the full repertoire of enzyme in this organism. Next, EMA takes this well-established list of enzymes and their reactions (the so-called stoichiometric matrix) to calculate the elementary flux modes (EFMs). The set of EFMs is unique and can represent any possible physiological situation in a cellular system. Aside from recovering canonical pathways, the EFM prediction approach also predicts a large number of unobserved potential pathways that emerge from a combination of active reactions in the network [72]. For this reason, the EFM approach is ideal to explore the richness inherent in a metabolic network and consequently to elucidate novel pathways [73,74]. To reconstruct a model for EMA, the set of directional metabolic reactions is a prerequisite. Defining the directionality of the reactions and finding all the optimal EFMs in a big reaction set is particularly challenging. Hence one needs a set of rules that can be followed to solve the problems with enzyme directionality and to have a tool that can break up big reaction sets into smaller connected networks that are easier to calculate using the EMA [75]. The same approach was used in the work on *A. fumigatus* [28]. In brief, using the KEGG database [76,77] and literature, the complete primary metabolism from *A. fumigatus* was modelled in a metabolic network, including major carbohydrate metabolism (glycolysis, pentose phosphate pathway, TCA cycle), lipid, fatty acid metabolism, nucleotide biosynthesis, amino acid biosynthesis and degradation, and fermentation pathways. The steady state of the system was computed using the YANA software package. To calculate condition-specific strengths of different metabolic fluxes, transcriptome data-sets were used as constraints to fit the metabolic model to estimate flux distributions [78,79] for optimal growth conditions and changes under biofilm conditions. The training procedure also involved algorithms (Gradient descent: BFGS—Boyden–Fletcher–Goldfarb–Shannon optimization method) that have been constantly developed further [80,81].

Other metabolism-based approaches include the inhibition of an enzyme that consumes a unique substrate. This results in the accumulation of the unique substrate which is potentially toxic to the cell. Moreover, the inhibition of an enzyme that produces a unique product may result in the starvation of the unique product, potentially crippling essential cell functions [82]. Such metabolically important enzymes are known as chokepoint enzymes in metabolic networks and could be used as potential drug targets. For instance, drug targets in *Plasmodium falciparum* were identified using chokepoint analysis [82]. These approaches become complicated, however, when identifying drug targets for eukaryotic pathogens.

Metabolic enzymes, as well as their regulators, are increasingly considered viable drug targets [83]. Often the pathogenic genes of *A. fumigatus* could be induced by conditions such as changes during invasion initiation [84], iron deficiency [85], and under hypoxia adaptation [86]. To reveal key enzymes involved in metabolic adaptation as prospective antimycotic targets, one needs to look at several related transcriptome datasets. Therefore, three GEO datasets were reanalyzed and the enzymes related to metabolism were identified [28]. Among the upregulated genes in reanalyzed transcriptome datasets, several genes were found to be important for *A. fumigatus* growth and hence were valuable antimycotic targets [28]. To access the genome or proteome, NCBI (https://www.ncbi.nlm.nih.gov/) or organism-specific portals can be used. For instance, AspGD [87] contains the omics data of several Aspergilli. For the reconstruction of the fungal metabolic network KEGG [76,77], Brenda [88], Reactome [89] re-evaluated by information from scientific literature represent the best resources. KEGG contains highly curated information that makes it the best one-stop option.

Metabolic models can also be derived on orthology based frameworks such as Raven and Merlin. Sequence similarity searches can be improved by using sensitive Blast parameters or reverse blast [90]. The recommended parameters for the best detection of orthologs as reciprocal best hits are the combination of soft filtering with a Smith–Waterman final alignment (the -F ‘‘m S’’ -s T options in NCBI’s BLASTP). Such considerations are important in order to avoid annotation errors which would mislabel the potential drug target [91]. Once the reaction network is created for FBA, one can select a number of metabolic modeling tools [92] with specific advantages and limitations. For EMA, we recommend the tool YanaSquare [78] and updated versions (https://www.biozentrum.uni-wuerzburg.de/bioinfo/computing/). It provides an easy interface for starters and experts. One can also use Metatool [93] for the EMA but its functionality is limited in comparison with YanaSquare. NCBI GEO [94] and ArrayExpress [95] are the major repositories of gene expression data which then can be analyzed with R [96] The built-in toll GEO2R can also be used to analyze the expression data deposited at the NCBI GEO database.

Important metabolic proteins were identified as potential antifungal targets by flux computations, for instance, the first enzyme of the riboflavin biosynthetic pathway GTP cyclohydrolase II (Rib1, AFUA_1G13300) was identified as potential target which possesses a capability as pace maker in the corresponding pathway [28].

### 3.3. Homology-Based Approach

Essential genes of pathogens can serve as drug targets if they do not have significant homology with host organisms. Database of Essential Genes (DEG) [97] contains genes reported in the literature to be essential for the organism tested under at least one condition for both the prokaryotes and eukaryotes. If the pathogen under investigation is not listed in the database, a Blast-based search with stringent e-value threshold <1 × 10^−10^ and a bit score ≥ 500, or orthology prediction can be used to identify the potential essential genes as we previously did for the pathogen *S. marcescens* [98].

Finding drug targets for eukaryotic fungal pathogens is more challenging than for bacteria (prokaryotes) because of the close human homologs of pathogen proteins. To prevent/reduce the possibility of cross-reactivity of potential drugs, it is important that the targets should not have similarity with human proteins. For such screening, Blast analysis with e-value cutoff <1 × 10^−5^ can be used to assess the similarity of network- or orthology-based on a non-redundant set of pathogen proteins with human sequences so that highly similar sequences can be discarded from further evaluation. In such cases, only the proteins that did not have hits below the e-value inclusion threshold are picked out as hosting non-homologous proteins of the pathogen.

Moreover, drug-resistant targets can be counter-screened from the putative list of drug targets by using homology search against ARDB, the Antibiotic Resistance Genes Database [99] and CARD, the Comprehensive Antibiotic Research Database [100]. This filter can limit the search space by screening out potential antibiotic resistance proteins based on Blast sequence similarity searches.

This approach identified cytochrome c oxidase family protein (AFUA_3G14440) and 50S ribosomal protein L30 (AFUA_4G10480) as essential genes whose products can be a potential antimycotic targets [28].

### 3.4. Ranking and Prioritization Criteria

The selected list of best potential drug targets can be scored and ranked according to various criteria, including: (i) removal of reactions catalyzed by true orthologs of human proteins; (ii) retaining of the ortholog of DEG based essential genes during the reduction process; (iii) characterization of the degree of these proteins (which were also present in interactome): an enzyme with high degree is more likely to be involved in multiple pathways and vice versa; (iv) selection of very few enzymes for any linear pathway stretch while considering the metabolites which are similar avoids combinatorial explosion during EFM calculation; (v) inclusion of pace-maker enzymes for long linear pathways; (vi) pathway annotation was done for this list and only the reactions which participate in primary metabolic pathway were further considered to find the drug targets in primary metabolism. Such criteria can be used to prioritize the preliminary drug target list of any pathogens.

Moreover, the R_humPDB_ score [101] can be calculated and the gene expression data can be mapped [102] over the preliminary target list to prioritize the drug targets.

$$R_{\mathrm{humPDB}=\log_{10}}\left(\frac{E_{\mathrm{BlastP}}\left[\mathrm{query}\;\mathrm{vs}\;\mathrm{human}\;\mathrm{proteome}\right]}{E_{\mathrm{BlastP}}\left[\mathrm{query}\;\mathrm{vs}\;\mathrm{DrugBank}\right]}\right)$$

A high R_humPDB_ score indicates the target has minimum similarity with human and has a close Protein Data Bank [103] structure template.

Additionally, the following criteria can be used to prioritize potential drug targets further: (i) highly expressed genes at many independent time-points can be given top priority (mean significant differential expression) over the genes highly expressed at less time points and so on; (ii) the final priority order can be decided based on the minimum of R_humPDB_ [101] and expression rank; (iii) if the enzyme participates in a pathway unique to the pathogen, this can be given higher rank; (iv) proteins can be ignored for which co-ortholog proteins are available, as this suggests that the same reaction can be catalyzed by alternative proteins.

Using the above-mentioned criteria and methods 64 targets were readily identified [28] including metabolic enzymes involved in vitamin synthesis, lipid synthesis, and amino acids, including 18 validated enzymes from literature, two validated and five examined in our own genetic experiments. Moreover, there are 38 further promising novel target proteins which are non-orthologous to human proteins, involved in metabolism and high ranked drug targets from these pipelines [28]. The predicted experimentally validated targets from the combined pipeline include enzymes such as chorismate synthase (ARO2, AFUA_1G06940) and chorismate mutase (AroC, AFUA_5G13130) which have shown a high antimycotic potential [104]. The pipeline identified two essential genes, the 50S ribosomal protein (AFUA_4G10480), and a cytochrome c oxidase family protein (AFUA_3G14440) that participate in oxidative phosphorylation, as good targets. Some of the targets are currently under experimental evaluation, such as 2,3-bisphosphoglycerate-independent phosphoglycerate mutase like conidial abundant protein (AFUA_3G09290) and urate oxidase (UaZ, AFUA_2G10520). Other potential targets identified include aspartate-semialdehyde dehydrogenase (Hom2, AFUA_3G06830) and phosphoribosyl-AMP cyclohydrolase (His4, AFUA_1G14570).

## 4. Toxic Intermediates and Fungal Toxins

Fungi are capable to catabolize and synthesize a variety of compounds such as plant hormones [105,106], pigments [107], antibiotics [108], and mycotoxins [109]. These compounds are valuable intermediates for fungal survival and can or are exploited for medicine and industry. Their identification can be done with several biochemical methods or by computational analysis of the metabolic pathways. Besides the metabolic pathways which are important for viability, fungi also possess ‘dispensable’ metabolic pathways, which describe pathways involved in the survival of the organism only under specific conditions, such as nutrient depletion or sporulation [110]. ‘Dispensable’ metabolic pathways can be activated by several environmental factors, such as temperature and pH, nutritional factors, including the presence of carbon and nitrogen sources and endogenous factors of the host [111]. Activated pathways result in the temporal production of intermediates that can be highly toxic to the fungal cell when they are accumulated. Detoxification of toxic intermediates can be done by several routes: (i) transport of the intermediates to another compartment of the cells (e.g., peroxisomes); (ii) assembling of the pathways that can utilize the toxic intermediates (metabolic channeling); and (iii) enzymatic lysis of the intermediates by enzyme (oxidoreductases and hydrolases) which are involved in the transformation of xenobiotic and natural products that are theoretically harmful for the fungi [112,113].

Toxins produced by fungi are able to evoke a toxic response also in mammals even when introduced in a low concentration. It was shown before that *Aspergillus* species can produce several toxic intermediates. Aspergillomarasmines were isolated from *A. oryzae*, *A. flavus*, and another fungal species [114] and have an inhibitory effect on angiotensin-converting enzymes and bacterial metallo-β-lactamases. The latter effect can defeat antibiotic resistance in Gram-negative bacteria and be a targeting molecule for the treatment of bacterial diseases [115]. Gliotoxin, the molecule of the epipolythiodioxopiperazines family, is produced by several fungal species including *A. fumigatus* and *C. albicans*. Gliotoxin is very toxic with the oral LD of 67 mg/kg with an immunosuppressive and proapoptotic effect on the mammalian cells [116,117]. Two toxicity modes of the gliotoxin were already reported: it acts as a redox-activator and generates the reactive oxygen species which can have a lethal effect on the cells, and it interacts with the proteins containing the free thiol groups [118]. Gliotoxin self-induces its biosynthesis by the activating of the *gli* cluster, however, the fungi are able to protect themselves against its harmful effect. The *gli* cluster consists of 13 genes in a positive feedback manner, where the transcriptional activator *gliZ* initiates the gliotoxin biosynthesis [119]. Self-protection of the fungus against the own gliotoxin is mediated by the enzyme GliT, a gliotoxin reductase [120]. The deletion of the *gliT* affects cell growth only in the presence of the gliotoxin and is not essential for its virulence [121]. Surprisingly, similar enzymes have been found in bacteria besides fungi, which can also facilitate the auto-protection against the epipolythiodioxopiperazines, such as holomycin production by *Yersinia ruckeri* and *Streptomyces clavuligerus* [122,123]. Several of the fungal intermediates have effective anti-bacterial properties, they can be used in the cosmetic and food industry. Moreover, fungi also compete against each other. A number of such toxin producing pathways including the polyketide synthetases may carry novel and useful antimycotic activities though this has mainly only been found for bacteria using genomics or metagenomics [124].

The direct effect of toxic intermediates on pathogens can be studied by analyzing metabolic pathways. The recent work of Ewald et al. [125] used a dynamic optimization approach to analyze the toxicity of intermediates and their effect on the control of the metabolic pathways. The authors analyzed linear metabolic pathways with different kinetic properties and toxicity of metabolites and looked at large-scale metabolic data sets of bacteria and fungi. Toxic intermediates are often present in such linear pathways; however, quick processing by downstream enzymes prevents their accumulation in the cell. Hence, a novel strategy prevents this fast processing of the toxic intermediate, for instance by blocking the downstream enzyme which then produces usually a non-toxic next product. Alternatively, the upstream enzyme may be elicited or activated and also this will then lead to higher levels of the next metabolite, the dangerous and toxic intermediate. These are two interesting novel strategies for antimycotics and the author describe targets in the ergosterol (cell wall synthesis) pathway which are new and increase such toxic intermediates when targeted [125].

The optimality principle can be used to reveal enzymes involved in essential pathways in fungi with sequence-similar protein counterparts in the host. Currently, several tools and databases have been developed for the study and modeling of the metabolic pathways that can be also used to describe the role of toxins in the fungi. Instead of the ‘classic’ databases such as KEGG (Kyoto Encyclopedia of Genes and Genomes, [77]), a focus on essential reactions can be an attractive alternative for targeting enzymes and metabolites for drug development. The species-specific essential reactions database (SSER) [126] is a database compiling biochemical and transport reactions that are necessary for the viability of the organism. SSER is using the flux balance analysis together with the manual curation of experimentally verified metabolic network that can facilitate the reconstruction of metabolic network and drug target analysis. The study of essential genes can be a promising step in order to discover the drug targets. It is necessary to note that the analysis of Aspergilli essential genes is challenging due to the poor efficiency of homologous recombination and thus incapability to study and reveal the essential genes. Nevertheless, identification of essential gene was already performed by several metabolic modeling studies. The *pNiiA*-CPR strategy described in the paper of Hu et al. [127] combined molecular technology together with the in silico genome-scale metabolic model of the drug screening with the focus on the erg11 gene. Ergosterol is the major sterol occurring in the fungi and protozoa and is essential for the resistance to azole drugs. The ergosterol biosynthesis pathway comprises about 20 genes. Interestingly, the comparative analysis of the members of the ergosterol pathway showed that several genes occur in duplicate, triplicate, and even in the quadruplicate among the *Aspergillus* sp., however, they occur only in one copy in the yeast. Interestingly, erg3 and erg11 belong to those multi-copy genes, and despite the differences in their sequences among the species, they are closely related among *Aspergillus* species [128]. The multiplicity of gene copies allows to control the arrangement and fluidity of the cell membrane and brings new adaptive strategies to drug development. Hu at al. demonstrated that inactivation of erg11A or erg11B genes does not lead to a clear phenotype in *A. fumigates*. The erg11 homologs can compensate the inactivation of the other gene, thus a drug treatment would have to effectively target both genes to inhibit the growth of the fungi [127]. An in vivo transposon mutagenesis system (random insertional mutagenesis) for searching of essential genes of *A. fumigatus* was introduced in the paper in Firon et al. [129], where then 20 previously uncharacterized essential genes could be identified. However, the limitation of this work is the generation of many gene deletions and various strain, this requires lots of time and is laborious. Thykaer et al. [130] has developed a purely computational approach for identification of essential genes, based on a genome-scale model, metabolome, and manual data mining. They identified 138 essential reactions, of which 33 belong to ‘artificial pathways’ that had been formed to model a pathway when the annotation of the genome was insufficient or the pathway was interrupted. Interestingly, these authors compared the obtained results with the work of Firon et al. [129], however, they matched only two proteins, guanylate kinase and S-adenosylmethionine decarboxylase, as they overlap in both studies. Moreover, they found one hit (mevalonate kinase) identified also in the work of Hu et al. [127].

Yet another antimycotic strategy is to use intermediates from other species that are specifically toxic for *Aspergillus*. The aflatoxin metabolic pathway can be inhibited by several proteins produced by Streptomyces. Aflastatins A and B, as well as blasticidin A constrain the production of aflatoxin by inhibition of very early intermediates of the aflatoxin pathway, resulting in glucose consumption and ethanol accumulation in the fungal body with harmful consequences for *Aspergillus* [131]. Similarly, DotA, a metabolite of Streptomyces, inhibits aflatoxin and sterigmatocystin production, but not the fungal growth. DotA uses an unknown mechanism to block the secondary metabolic pathways, where interestingly, the pathways resulting in the production of kojic acid and yellow pigment are markedly upregulated [132].

Fungi need to utilize also several metals in their enzymes which act also as microbial toxins, including copper and iron [133]. These metals are two-facing players in the fungal body: they are essential coenzymes for enzymes (e.g., Sod1, CrpA, CmtA, AceA); however, they are also a catalyst in toxic ROI-generating Fenton chemistry [134]. Hence, the iron-acquisition system is a promising target for drug design because the proteins importing the ferri-siderophores were found only in the fungi, thus they do not affect the human cells. Adaptive behavior of *A. fumigatus* in the excess or lack of the metals can be investigated through several approaches. Linde et al. [135], in his pioneering work, designed the top-down method using the gene expression time-series data to build the regulatory network and predict the interactions between the transcription factors and target genes to study the iron regulation. However, this method requires a large set of data and parameters, which can be incomplete and thus this approach has limitations. Brandon et al. [136] applied a stochastic Boolean network model analysis and simulated the iron acquisition and oxidative stress response including several phenotypes of knockout strains. Taken together, these data demonstrate that fungi can effectively respond to metal starvation and oxidative stress at the transcriptome and proteome level. A comprehensive study recently published by Kurucz et al. [137] involved trancriptomic analysis, functional annotation, and proteomic profiling to reveal response of *A. fumigatus* to iron depletion. Interestingly, it was found that although the pathways in human and fungi are highly conserved across taxa, several specific differences can lead to successful antifungal treatment [138,139].

These techniques are well established and in silico screens on new antifungal targets are hence pursued with intensity, for instance regarding *Candida albicans* [140], while concrete validation is of course rather time consuming and lagging here somewhat behind while also sometimes invalidating the in silico result [141]. Another interesting alternative is hence repurposing of drugs, easily but not perfectly achieved using the STITCH database [142]. To give a concrete antifungal example, (+) (S) Abscisic Acid was recently identified as a new antifungal by ligand-based virtual screening of homology models for fungal chorismate mutases and a pharmacophore model derived from a transition state inhibitor [143]. Promising structure-based techniques are hence described next.

## 5. Validating and Verifying the Antifungal Target Quality and Antimycotic Drug Screening

### 5.1. Target Identification

The demand for new antifungal drugs is increasing with the increased incidence of fungal infections. However, most of the antimycotic medications fail in trials for one of two reasons: either they are not effective or they are not safe. Therefore, one of the key steps in developing a new drug is target identification and validation [144]. Potential targets are most often proteins. Genes, RNAs and for instance cell wall components are usually targeted via the enzymes involved in their synthesis. A good target needs to be “druggable”—accessible to drug molecules, with a therapeutic effect upon binding, safe and without cross-reaction with other drugs or cells. Data mining with implementation of bioinformatics tools helped to identify, select, and prioritize potential disease targets [144,145]. This includes analysis of gene expression data, proteomics data, transgenic phenotyping, and compound profiling data. Identification approaches also include examining mRNA/protein expression levels to elucidate their role in disease initiation and progression.

### 5.2. Target Validation

**Figure 4:**
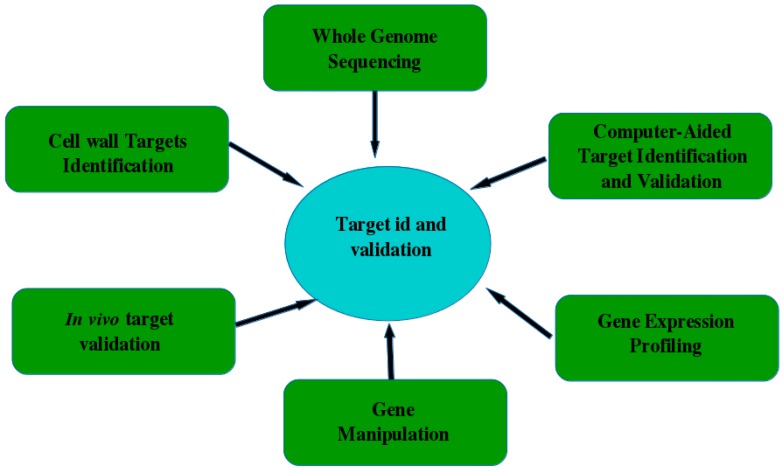
Schematic representation of some examples for target identification and validation.

When the predicted target is identified, its accurate exploration is necessary. Validation techniques vary from in vitro tools up to the use of animal models and consideration of an important target in diseased patients [144]. The most popular methods for the target validation are whole genome sequencing [146,147,148]; computer-aided target validation that uses simulation to identify therapeutic targets [149]; gene expression profiling, gene manipulation [150]; in silico analysis of gene knockouts [151]; and finally in vivo proof models, which can quickly provide key data for target genes [152]. Figure 4 shows some examples of target identification and validation methods.

### 5.3. Protein Structure-Based Drug Design Strategies

Structure-based drug design (SBDD) is one of the most successful strategies for computation drug design based on the three-dimensional (3D) protein structure [153,154,155]. The 3D structural data can be obtained either experimentally using methods such as X-ray crystallography, NMR spectroscopy, cryoelectron microscopy, or by computational homology modeling [155,156] in the case that the 3D structure is not available. Homology modeling can be performed by several web server tools such as SWISS-MODEL [157], I-TASSER [158], or by specialized software such as MOE [159] or Modeller [160]. For software-based modeling, template crystal structures can be retrieved from the Protein Data Bank (PDB) (https://www.rcsb.org) [103] and/or Cambridge Structure Database [161]. An essential point in structure-based drug design studies is an understanding of the spatial and energetic aspects that influence the binding affinities of drug target complexes. The availability of macromolecular structure enables a detailed examination of binding cavities on their residual compositions, shape, as well as electrostatic properties.

SBDD is a stepwise process. First, in silico tools are implemented to identify potential ligands for the known protein structure, which is followed be the synthesis of the promising compounds. Next, biological properties—such as potency, affinity, and efficacy—are evaluated by diverse experimental measurements. Once a ligand–receptor complex has been established, further steps towards molecular modifications with an intention to increase the affinity of new ligands for the binding site are taken. However, the flexibility of the target receptor is an essential aspect one has to take into consideration while constructing new drugs since the conformational change of a protein can occur upon ligand binding. Computational techniques such as flexible docking and molecular dynamics (MD) are quite useful in handling a flexibility issue [155,162,163]. Molecular docking predicts the conformation of a ligand within a binding pocket driven by binding energetics and affinity [164]. It can be applied to generate a series of docking samples when no crystallographic structure of the target is available [165]. In addition, MD can also be used to estimate the stability of a ligand–receptor complex obtained by molecular docking [155,166]. This is usually achieved by comparing the root mean square deviation (RMSD) value of ligand pose obtained from docking simulation with the one obtained from MD [167]. If the RMSD value of ligand, calculated from MD simulation, deviates more than the one obtained from docking, then the predicted protein–ligand complex can be considered unstable.

**Figure 5:**
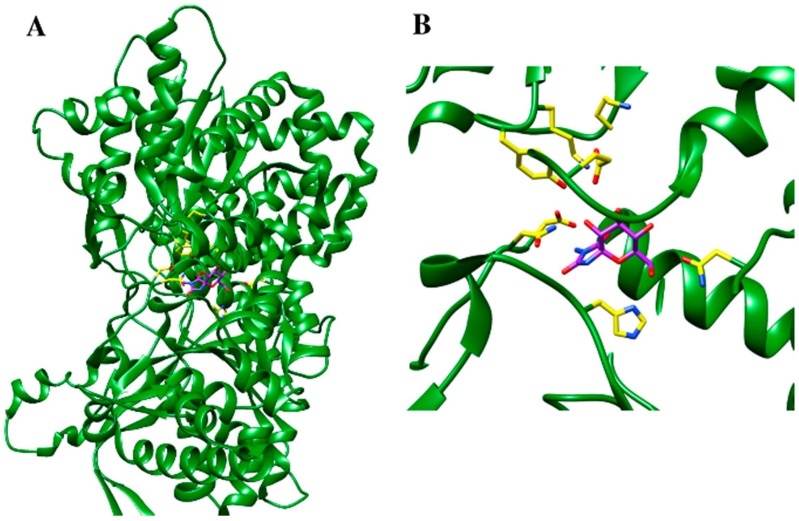
(**A**) Glycogen phosphorylase complex with its inhibitor (3-amino-8,9,10-trihydroxy-7-hydroxymethyl-6-oxa-1,3-daza-spiro[4.5]decane-2,4-dione). (**B**) Magnified representation of the binding site with inhibitor. The enzyme is represented as a secondary structure and shown in green, residues at catalytic site as well as an inhibitor are represented as ball and stick and highlighted in yellow and purple, respectively [168].

An example of structure-based drug design for a metabolic enzyme is a complex of glycogen phosphorylase (PDB accession number 1FTQ) with its inhibitor 3-amino-8,9,10-trihydroxy-7-hydroxymethyl-6-oxa-1,3-daza-spiro[4.5]decane-2,4-dione [168] as is depicted in Figure 5. In this particular case the catalytic site of the enzyme was examined and the new inhibitors were constructed by taking into account an information of the binding site as well as the chemical structures of the already known glycogen phosphorylase inhibitors—spirohydantoin and *N*-acetyl-β-d-glucopyranosylamine [168]. Based on these results, the suggested step can be the development of new fungus-specific inhibitors of such central metabolic enzymes, minimizing side-effects in the host.

Fragment-based drug design is another approach for rational drug design [169]. It considers a combination of small molecules with poor binding ability to the active site that yields to a new compound with higher potency than the separate pieces. This can be achieved either by incremental growth of the fragment inside the ligand binding pocket while interacting with the surrounding residues in the pocket or by merging different fragments to achieve an optimal interaction with the residues of the active site. Usually, these fragments have a molecular weight less than 250 Da and LogP less than 3 [169]. This computational strategy in drug design originates from the late 80s with an implementation of computational tools such as GRID [170], MCSS [171], LUDI [172], SPROUT [173], SuperStar [174], and MUSIC [175]. Recent approaches in fragment based drug design include LigBuilder 3 [176], AutoGrow [177], and MOE (Molecular Operating Environment 2013 [159]).

In order to select promising compounds for a given target, a virtual screening (VS) of large chemical databases is a quite powerful method in terms of saving time and budget [155]. There are two main types of VS: ligand- and structure-based virtual screening (LBVS and SBVS, respectively). LBVS is based on the investigation of molecular features collected from compounds that are active. In general, a set of common characteristics for a number of such compounds is identified, which are further employed as molecular filters. These database filtering methods are used to select compounds for experimental evaluation and to reduce the chemical space to be explored in further screening steps. Another strategy of LBVS is to generate a pharmacophore model. This implies the use of basic structural features, collected from known active ligands that are necessary to produce biological effects. Several programs [155] such as Hiphop, HypoGen, GASP, MOE, PharmaGist, GAMMA, and LigBuilder are available for pharmacophore modeling.

SBVS consists of several steps: molecular target preparation, compound database selection, molecular docking, and post-docking analysis. In SBVS, the prepared compound database is docked into a binding site of a selected target [178]. Besides the prediction of the binding pose, SBVS provides a ranking of the docked molecules, which is later used as a criterion for selecting promising drug candidates. The selected compounds are then evaluated experimentally for their safety and biological activity. The most frequently used chemical databases for the virtual screening are PubChem, Zinc [179], ChemSpider [180], and DrugBank [181].

To quickly assess an information related to protein–protein interactions and availability of existing drugs to modulate such an interaction network, a new drug-minded protein interaction database (DrumPID) has been developed by Kunz et al. [182]. The search strategy of the server allows users to access any relevant information from multiple query windows. For example, one can search for generic drug name, target protein, chemical structure (SMILES annotation), as well as study and compare targets and interactions between different drugs. The output of the database is a comprehensive overview of the chemical characteristics, involving metabolic pathways, orthologous groups, and other natures of the drugs [182].

Such approaches are well established, and some recent efforts regarding antifungals include prediction of antifungal peptides using a support vector machine (available as a server; http://webs.iiitd.edu.in/raghava/antifp [183]) and docking, design as well as synthesis and antifungal activity study of novel triazole derivatives against *A. fumigatus* with improved activity compared to fluconazole [184].

A comparative novel approach tries to model host–pathogen interactions directly in silico applying game theory [185]. In this way, a wide-range of parameters during the infection can be sampled by different rules and macrophage immune defense against *Candida albicans* delineated. As it allows to quantify how optimal the immune strategy is or where it is lacking, this has the potential to point to therapeutic options e.g., in immunocompromised patients including delineation of molecular targets such as factor H and modifications of it [186].

## 6. Concluding Discussion

### Methods and Their Perspective

We introduce here general bioinformatics methods for identification of unique fungal pathways that allow us to screen efficiently for safe new drug targets and design new antimycotics. We describe several different bioinformatics pipelines, by which we can find numerous new fungal targeting strategies. In general, one should consider the biology of the fungal species, compare virulence and pathogenesis to spot interesting fungal-specific differences. For this, an evolutionary perspective is important, too. For instance, *A. fumigatus* is a versatile survivor, usually with a saprophytic life style and only causes disease, if there is an immunocompromised person infected. Thus, the only real ‘weapon’ it uses are toxins such as gliotoxin, developed against amoeba—its natural enemy that exists in its usual ecosystem. The adequate strategy to cope with the fungal infection is to take a closer look at metabolic pathways, enzymes involved in the synthesis and maintenance of the cell wall and regulation. The utilization of bioinformatics methods can produce data useful for the design of new drugs. Another important process that we have to consider is the virulence and the selection for the virulence factors, which are usually not present in the fungi. Pathogenic fungi such as *Aspergillus* can inhabit disparate niches and their ‘virulence’ occurs when a combination of host and fungus takes place. This happens when there are factors favoring high adaptability in low nutrient conditions. For instance, pathways supporting *Aspergillus* during iron deficiency help the fungus also to prosper in the lungs of immunocompromised patients (*A. fumigatus*) as well as to reach new human niches (*C. albicans)*. Similar considerations apply for *Candida auris* [187].

In this paper, we started from the well-known and established standard methods to identify fungal unique pathways—such as genome annotation, biochemical databases, and domain analysis—to identify fungal-specific adaptations that can be targeted. The reader learns a couple of pipelines and can be assured that many bioinformatics laboratories use such and similar methods to identify drug targets in fungi or bacteria. Here, we tried to give instructive examples and useful methods to clarify different approaches how novel targets can be identified by bioinformatics.

Another hot topic of fungal target identification by bioinformatics is network biology. Network biology approaches can be established by several different methods and databanks, which combine the data of various omics studies. This comparison of host and pathogen interaction networks is called ‘interactomics’. We provide from this several example of successful identification of novel host–fungal interactions that open new alleys and drug options to treat fungal infections. The network-based approach has very good theoretical foundations, is reliable and confidential. However, as detailed in the corresponding chapter, the success depends on the reliability of the interaction data. Since databases contain a number of such data, the extrapolation to a new organism and fungus (e.g., *A. fumigatus*) is inherently risky if not supported by experimental analysis and, at the very least, additional bioinformatics analysis of the postulated interaction. A particularly good strategy is to combine such predictions with metabolic modeling. If such predictions can be refined and combined with in vivo testing and experimental follow-up, the success rate is high.

The European Union sponsored project “AspMetNet” based on the initiative EraNet successfully utilizes the bioinformatics prediction combined with metabolic and signaling modeling as well as experimental verification to better cope with infectious disease, mainly focused on the species *A. fumigatus*. Thus, the genome-wide analysis of protein interaction networks is certainly an exciting new perspective that was not possible before and, which allows unprecedented general and thorough screening of all fungal pathways for new antimycotic targets.

To put such new bioinformatics approaches into perspective, we also introduce a fresh look at toxins as antifungal targets. Interestingly, first of all, fungal toxins—such as gliotoxin—traditionally are regarded only as products of the fungus. However, as mentioned above, there is a clear potential to use such toxins as the basis for novel antifungals thanks/due to their lethal effect when are accumulated in the fungal cells. Secondly, after a theoretical analysis, we concluded that not only such toxins, but even toxic intermediates, in general, pose a substantial risk for any microorganism and fungi, in particular. Herein, we give concrete examples including the ergosterol pathway that by suitable manipulation (in the manner of the drug blocking the subsequent enzyme or drug enhancing the toxic intermediate producing enzyme) is new space for novel antimycotics against which it is difficult to develop resistance.

Beyond the typical techniques cited, there is of course ongoing research, too. In such studies, experimental validation is critical, for instance Fungal sterol C22-desaturase did not turn out as an antimycotic target after extensive experimental testing [141]. Moreover, one should keep on mining natural substances. Thus it was recently shown that crotalicidin from rattlesnake displays antifungal activity against clinical and standard strains of *Candida* [188].

Finally, in the last chapter, we sketch the path for the next step, the design of novel drugs which can bind to or inhibit identified targets. We describe several approaches, such as fragment-based drug discovery, or “good old strong work horses” such as docking and molecular dynamics including different in silico virtual screens that can target, identify and analyze the drug candidates.

We are convinced that, beside the approaches mentioned in this paper, the space for novel strategies against antimycotics is far bigger than one would think as a non-expert and we hope we can stimulate research in this direction.

## 7. Future and Outlook

Antibiotics are inexpensive, well established, but not one hundred percent effective molecules which are the golden standard to treat bacterial diseases. The same applies to antimycotics. As the return of investment in new antimycotics is low for companies, the drug development pipelines are running out. However, there is always a need for new antimycotics as there are new emerging fungal diseases (for example *C. auris*) and, above all, fungi species become resistant. Bioinformatics can help here to rapidly provide insights from the genome sequence. For instance, a current sequencing effort on *C. auris* species compares hospital isolates regarding virulence factors, pathogenicity islands, as well as resistance plasmids and toxins.

Apart from a financial benefit for pharmaceutical companies, the scientific outlook is also promising. As discussed above, there are many ways to examine *A. fumigatus* metabolism, cell wall networks, and regulation. The current antimycotic drugs target mainly fungal differences comparing human and fungal proteins. With the help of bioinformatics, we can identify these differences even better and devise novel strategies to best target them. Moreover, we can screen huge amount of potential drug candidates to select best leads for further optimization according to specific pharmacological needs.

## Figures and Tables

**Table 1:** Common fungal diseases in animals. Sources: https://www.msdvetmanual.com and https://www.britannica.com.

Host Species	Agent	Diseases	Description	Treatment
Honeybee (*Apis mellifera*)	*Ascosphaera apis*	Chalkbrood	Fungus affects the gut of larvae. Outcompetes larvae for nutrition and turns the larvae into “chalk-like” mummies. Very infectious.	Apiguard, thymol-based treatment
Honeybee (*Apis mellifera*)	*A. fumigatus*, *A. flavus*, *A. niger*	Stonebrood	The fungus causing the green or yellow mummification of larvae. Very infectious but not common.	No treatment
All mammals, birds	*A. fumigatus*, *A. flavus*, *A. nidulans*, *A. niger*	Aspergillosis, Keratomycosis (horses)	Fungal infection attack mostly disease-weakened pets, disease or drug therapy is possible. The nasal and pulmonary form is most typical.	Ketoconazole, Itraconazole, Fluconazole
Dogs, horses (rarely cats)	*Blastomyces dermatitis*	Blastomycosis	Systematic mycosis described in humans, dogs, and horses, endemic in North America.	Amphotericin B and ketoconazole (dogs), no treatment for horses
Horses, mules, donkey, cattle	*Histoplasma farciminosum*	Epizootic Lymphangitis	The chronic fungal disease causes inflammation and suppuration of the cutaneous and subcutaneous lymphatic vessels and glands.	No treatment (only surgical excision of all affected nodes)
All mammals, birds	*C. albicans*, *C. fumata*	Candidiasis	Mucocutaneous disease infecting mostly the nasopharynx, gastrointestinal tract and external genitalia. Often occurs in birds, in cats candidiasis is rare. Cause of arthritis in horses and mastitis in cattle.	Amphotericin B, Fluconazole, Chlorhexidine (birds)
